# Rapid reduction of malaria following introduction of vector control interventions in Tororo District, Uganda: a descriptive study

**DOI:** 10.1186/s12936-017-1871-3

**Published:** 2017-05-30

**Authors:** David W. Oguttu, Joseph K. B. Matovu, David C. Okumu, Alex R. Ario, Allen E. Okullo, Jimmy Opigo, Victoria Nankabirwa

**Affiliations:** 1 Uganda Public Health Fellowship Programme-Field Epidemiology Track, P.O. Box 7072, Kampala, Uganda; 2 Tororo District Health Office, Tororo, Uganda; 3 National Malaria Control Programme, Ministry of Health, Kampala, Uganda; 4 School of Public Health, Makerere University, Kampala, Uganda; 5 Centre for Intervention Science in Maternal and Child Health, Centre for International Health, University of Bergen, Bergen, Norway

**Keywords:** Malaria, Incidence, Reduction, Vector, Control

## Abstract

**Background:**

In 2012, Tororo District had the highest malaria burden in Uganda with community *Plasmodium* prevalence of 48%. To control malaria in the district, the Ministry of Health introduced universal distribution of long lasting insecticide-treated nets (LLINs) in 2013 and added indoor residual spraying (IRS) in 2014. This study assessed malaria incidence, test positivity rates and outpatient (OPD) attendance due to malaria before and after vector control interventions.

**Methods:**

This study was based on analysis of Health Management Information System (HMIS) secondary malaria surveillance data of 2,727,850 patient records in OPD registers of 61 health facilities from 2012 to 2015. The analysis estimated monthly malaria incidence for the entire population and also separately for <5- and ≥5-year-olds before and after introduction of vector control interventions; determined laboratory test positivity rates and annual percentage of malaria cases in OPD. Chi square for trends was used to analyse annual change in malaria incidence and logistic regression for monthly reduction.

**Results:**

Following universal LLINs coverage, the annual mean monthly malaria incidence fell from 95 cases in 2013 to 76 cases per 1000 in 2014 with no significant monthly reduction (OR = 0.99, 95% CI 0.96–1.01, P = 0.37). Among children <5 years, the malaria incidence reduced from 130 to 100 cases per 1000 (OR = 0.98, 95% CI 0.97–1.00, P = 0.08) when LLINs were used alone in 2014, but declined to 45 per 1000 in 2015 when IRS was combined with LLINs (OR = 0.94, 95% CI 0.91–0.996, P < 0.0001). Among individuals aged ≥5 years, mean monthly malaria incidence reduced from 59 to 52 cases per 1000 (OR = 0.99, 95% CI 0.97–1.02, P = 0.8) when LLINs were used alone in 2014, but reduced significantly to 25 per 1000 in 2015 (OR = 0.91, 95% CI 0.88–0.94, P < 0.0001). Malaria test positivity rate reduced from 57% in 2013 to 30% (Chi = 15, P < 0.0001) in 2015. Slide positivity rate reduced from 45% in 2013 to 21% in 2015 (P = 0.004) while RDT positivity declined from 69 to 40%.

**Conclusions:**

A rapid reduction in malaria incidence was observed in Tororo District following the introduction of IRS in addition to LLINs. There was no significant reduction in malaria incidence following universal distribution of LLINs to communities before introduction of IRS.

## Background

Despite scale up of interventions geared towards control and eventual elimination of malaria, the disease is still endemic in 91 countries worldwide with 3.3 billion people at risk of developing malaria in any given year. In 2015, 212 million cases of malaria occurred globally resulting into 429,000 deaths of which 92% were in Africa [1]. High malaria burden has been identified as one of the causes of poverty in many developing countries [2]. However, with scale-up of interventions in most endemic countries, global reduction of malaria burden has been reported [1].

Uganda is among the 15 countries most affected by malaria in the world [3, 4]. The national malaria indicator survey of 2009 reported that 45% of Ugandan children aged below 5 years carried malaria parasites. According to the Ministry of Health (MoH), malaria accounts for 25–40% of outpatient visits in public health facilities and is responsible for half of inpatient paediatric deaths. *Plasmodium falciparum* is the predominant species of malaria in Uganda [5, 6]. The major malaria parasite vectors in the country are *Anopheles gambiae* complex and *Anopheles funestus* [7]. Malaria transmission in Uganda is perennial with two peaks in April to June and October to December, corresponding to the rainfall seasons during which the vector biting density is high [8, 9]. The highest global entomological inoculation rate has been reported in Uganda [8]. The country is among others still in the attack stage of malaria control [9, 10]. The goal of Uganda’s malaria control policy is to reduce morbidity and prevent mortality attributable to malaria [11]. The US Present’s Malaria initiative supported strategies including proper case management of non- complicated cases using artemisinin combination therapy (ACT), intermittent preventive therapy in pregnancy (IPTp), Integrated Community Case Management (ICCM), distribution of long-lasting insecticide-treated nets (LLINs) to communities and indoor residual spraying (IRS) in epidemic prone and hyper-endemic districts have been scaled up in Uganda. In 2013, the MoH achieved universal distribution of LLINs to all communities by offering a free bed net to every two people in a household. In 2006, IRS was started in 10 districts of Northern Uganda as a complementary strategy to LLINs and ACTs to achieve faster reduction of malaria incidence in the region [12]. In 2014, IRS was scaled up to more 14 high malaria prevalence districts in Northern and Eastern Uganda [13].

Evaluating effectiveness of such malaria interventions over time often requires well designed studies such as controlled randomized trials [14–16]. However, such studies are usually not feasible due to inadequate resources. Health Management Information System (HMIS) was introduced to collect routine surveillance data in health facilities useful for monitoring and evaluation of interventions [17]. The HMIS captures all data required to determine all malaria indicator variables such as incidence and test positivity rate. Since the roll out of the District Health Information Software, version 2 (DHIS2) in 2012 [18], routine malaria surveillance has improved. All public and high volume private health facilities report malaria cases weekly to the MoH using HMIS 105 form. With improving completeness of reporting, available routine surveillance data can be analysed and used by districts to monitor trends of malaria incidence and evaluation of malaria interventions [19]. However, due to inadequate malaria epidemiology expertise in districts [20, 21], these data are not fully utilized. The study analysed surveillance data of a high malaria endemic district which implemented universal distribution of LLINs in 2013 and advanced to IRS in combination with LLINS in December 2014. The aim of this study was to use routine malaria surveillance data to assess change in malaria infection using incidence, test positivity rates and outpatient (OPD) attendance due to malaria as key indicators at three time-points (when there were no vector control interventions; when LLINs were used alone and when LLINs were used in combination with IRS). Malaria incidence is a measure of the rate of new malaria cases in the population and an important indicator of the frequency of the disease transmission.

## Methods

### Study area

Tororo districts is located in eastern Uganda. It is composed of 17 rural sub-counties, 2 town councils and two municipal divisions. The district has a population of 526,378 (National Census 2014). The Uganda malaria indicator survey of 2009 [22] and earlier surveys [23] ranked Tororo among the highest malaria burden districts with annual entomological inoculation rate of 591 infective bites per person per year [23]. The district geographical features and economic activities favour breeding of malaria vectors. Wetland rice growing is one of the popular agricultural activities leading to high transmission of malaria [24]. During rainy months of the year, rice gardens flood and hold water for long periods, providing potential breeding sites for *Anopheles* mosquitoes. *Anopheles gambia* is the major malaria vector. In rural villages, most people live in grass thatched houses which favour indoor resting and biting of malaria vectors [25]. Entomological surveys have described Tororo as having a very high population of *Anopheles* mosquitoes feeding and resting inside inhabited grass thatched houses with a rate of 160 bites per person per night and a high annual entomological inoculation rate [5].

Tororo district has 5 hospitals and 56 lower level health facilities located in sub-counties. All these health facilities submit weekly and monthly malaria surveillance data to the district through HMIS. During 2012–2015, the 61 health facilities recorded a total of 2,727,850 patients as new attendees in OPD. Small health facilities and hospitals capture all new clinical cases in OPD registers. Despite the roll out of the malaria test and treat policy by the Uganda Ministry of health in 2012, its implementation in Tororo district was slow because of inadequate supply of test kits. Health workers in most facilities continued to treat and report presumptive malaria cases [26–28] based on a standard case definition (Uganda clinical guidelines).

### Malaria case and outcome variable definitions

According to the Uganda HMIS, both presumptive and confirmed malaria cases are captured and summed up to give total malaria cases. Confirmed malaria cases were patients whose blood samples tested positive by RDT or blood slide using microscopy. On the other hand, presumptive cases were patients who presented with fever and were clinically diagnosed (Uganda clinical guidelines 2012) and treated for malaria by health workers without testing. The malaria testing rate in Tororo district health facilities gradually increased from 30% in 2012 to 85% in 2015. A sum of presumptive and confirmed cases was reported as total malaria cases per month in HMIS. Due to the low testing rates, total malaria cases were used to estimate incidence and confirmed cases to estimate the test positivity rates. Incidence was defined as the number of new malaria cases per 1000 population in a specified period. Monthly malaria incidence was an estimate of new malaria cases in the population per month. The annual mean monthly malaria incidence was the average proportion of new malaria cases in the population every month of each year. It is an estimate of the mean frequency of new malaria cases in the population per month. Slide positivity rate was defined as the percentage of tests which were positive for malaria out of the total tests done using microscopy or rapid diagnostic test kits. It estimates the prevalence of confirmed malaria cases among the tested patients.

OPD attendance due to malaria was calculated as the percentage of patients diagnosed by laboratory and clinically to have malaria out of the total patient attendance recorded in OPD registers of health facilities. It is an estimate of the extent to which malaria causes illness in the population leading to high expenditure of district resources to treat malaria.

### Study design

This was an observational retrospective analysis of aggregated routine surveillance data of malaria reported in HMIS from 2012 to 2015. The reporting rate of malaria cases by Tororo district health facilities through HMIS improved from 70% in 2012 to 99% in 2015. However, in 2012, data reporting through DHIS2 started in the second half of the year.

### Malaria control interventions

Before 2013 treatment of malaria cases in health facilities was the major malaria control strategy used. Clinical diagnosis using standard case definition (Uganda clinical guidelines) and treatment of presumptive malaria cases was the commonest practice. The roll out of the malaria test and treat policy in 2012 (Ministry of Health guideline) was not achieved until 2015 when the testing rate increases to 85% (Uganda HMIS). Both presumptive and confirmed cases were reported as total malaria cases in HMIS. Ownership of LLINs in communities was below the standard coverage recommended by the WHO [29], with about 50% of the households having a bed net [22]. In December 2013, Tororo district achieved universal (98%) coverage of LLIN distribution [30] to all communities, giving one free bed net per two individuals in a household. In December 2014, integrated vector management [31] was started by introducing IRS in addition to LLINs [32]. The first round of IRS with a carbamate insecticide (bendiocarb) achieved 85% coverage and in June 2015; a second round covered 95% of all residential houses in the district. Malaria vectors in Uganda are susceptible to bendiocarb [33, 34]. By the time of this study other community-based malaria control strategies such as the Integrated Community Case Management of malaria (ICCM) had not been implemented in Tororo district. In ICCM strategy voluntary village health teams are supplied with malaria RDTs to confirm the disease in febrile children in communities and treat them using ACT.

### Data source

Population data were obtained from the National Census report 2014 and the planning department of Tororo district. The routine malaria surveillance data reported passively through public and high volume private health facilities were accesses from the data base. Weekly and monthly malaria case reports are made to the MoH by all health facilities through HMIS. The district reports (unpublished) show the monthly reporting rate of public health facilities through DHIS2 has increased from 70% in 2012 to 99% in 2015. From health facilities, malaria data are reported by age (0–4 and ≥5 years) and sex (male or female). The number of new malaria cases recorded in out-patient (OPD) registers includes those tested in the laboratory using rapid diagnostic kits (RDTs) or microscopy and cases diagnosed and treated clinically based on the Uganda clinical guidelines. Using DHIS2, the district data are accessed by the Ministry of Health epidemiologists and partners with access rights.

### Data analysis

Analysis of data was done to estimate change in the magnitude of malaria using three malaria burden indicator variables focusing specifically on incidence, test positivity rates and percentage of reported malaria cases in OPD [9, 35]. Monthly data on out-patient (OPD) attendance, reported malaria cases and test positivity rates from 2012 to 2015 were accessed from the HMIS using the District Health Information Software 2 (DHIS2). Data were down loaded into Microsoft Excel.

Incidence was calculated to obtain the number of new malaria cases reported per 1000 population. The proportion of malaria cases in OPD per year was calculated as a percentage of the number of malaria cases recorded in a year out of the total OPD attendance. Slide and malaria RDT positivity rates were calculated as a percentage of the positive tests divided by the total number of patients tested. Line graphs to illustrate monthly malaria incidence were made. Change in malaria incidence following universal distribution of LLINs and IRS in comparison to previous years was described. Chi square test for trend was used to assess significance of the observed annual changes in malaria indicators following introduction of vector control interventions. Analysis of the trend of monthly changes in malaria incidence following universal distribution of LLINs and after introduction of IRS was done using logistic regression.

## Results

### Reduction in annual malaria incidence

Table 1 shows the change in annual malaria incidence in Tororo District before and during vector control interventions. Before large-scale vector control interventions were initiated, malaria incidence was 910 per 1000 population in 2012 and 1135 per 1000 in 2013. The district to introduce LLINs for pregnant mothers in the latter half of 2013 followed by universal LLINs in 2014. Following this intervention, malaria incidence dropped to 910 per 1000 population. Introduction of IRS alongside LLINs was followed by a reduction of malaria incidence by 52% to 434 cases per 1000 population in 2015. Implementation of vector control interventions was followed by significant reduction in annual malaria incidence in the district from 2013 to 2015 (Chi square = 1052, P < 0.001).

**Table 1:** Change in annual malaria incidence in Tororo District before and during vector control interventions 2012–2015

Year	Vector control intervention	Malaria cases	Incidence per 1000	% Facilities reporting in form 105.1
2012	No vector control	430,537	910	70
2013	LLINs for pregnant mothers	439,946	1135	95
2014	Universal LLINs	377,701	910	97
2015	LLINS + IRS	193,950	434	99

### Trend of annual mean monthly malaria incidence

**Fig. 1:**
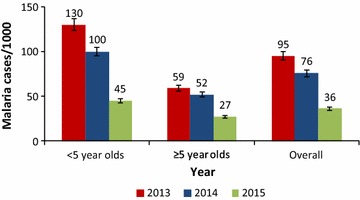
Change in annual mean monthly malaria incidence in Tororo District, 2013–2015. The figure shows the overall and age-stratified annual mean monthly malaria incidence before intensified vector control interventions were introduced (2012–2013), during the period when universal coverage of communities with LLIN was used alone in 2014 and when LLINs were combined with IRS in 2015. Overall, there was significant reduction in annual mean monthly malaria incidence from 95 in 2013 to 36 cases per 1000 in 2015 following introduction of integrated vector control interventions (chi = 27.5, P < 0.0001). Among children <5 years, there was a more significant reduction in annual mean monthly malaria incidence (Chi square = 43.9, P < 0.0001) compared to individuals aged 5 years or older (chi = 12.0, P = 0.0005) during the same period

Among <5-year-olds, mean monthly incidence was 119 per 1000 in 2012; increased slightly to 130 in 2013 but dropped to 100 per 1000 when LLINs were introduced. When IRS was applied in combination with LLINs in 2015, malaria incidence dropped by 55%. A similar reduction occurred among those aged 5 years and older (Fig. 1).

### Trend of monthly malaria incidence

**Fig. 2:**
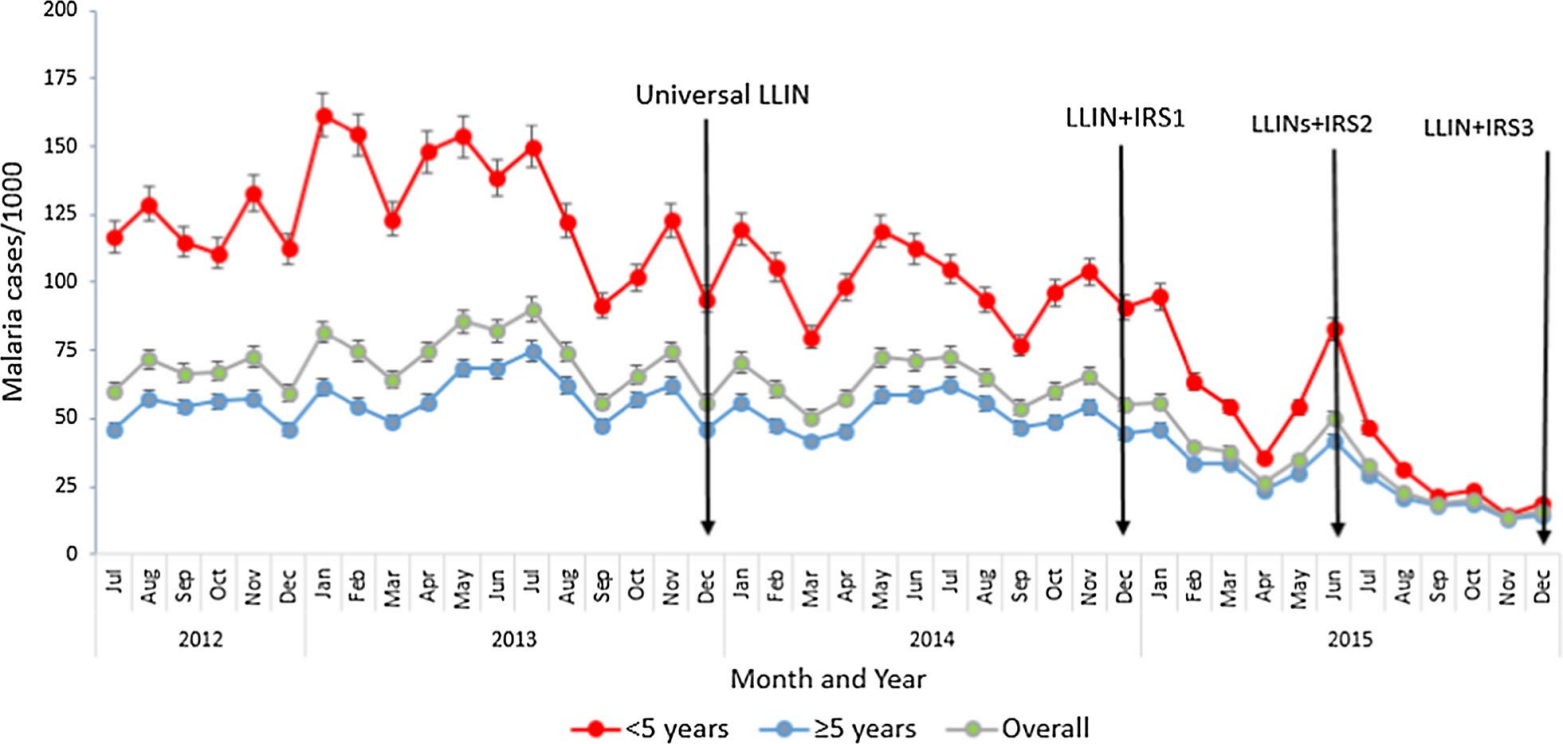
Trend of monthly malaria incidence in Tororo District before and following vector control interventions 2012-2015. The figure shows overall and age stratified changes in monthly malaria incidence before and following intensified vector control interventions. Logistic regression analysis of monthly incidence trend showed no significant reduction from January to December 2014 when LLINs were used without IRS (OR = 0.99, 95% CI 0.96–1.01, P = 0.37). Combining LLINs with IRS in 2015 was followed by significant reduction in monthly malaria incidence during January to December 2015 (OR = 0.89, 95% CI 0.87–0.92, P < 0.05). There was no significant reduction of monthly malaria incidence among under fives (OR = 0.98, 95% CI 0.97–1.00, P = 0.08) and the ≥5 age individuals (OR = 0.99, 95% CI 0.97–1.02, P = 0.8) from January to December 2014 when LLINs were used without IRS. On the other hand, introduction of IRS to complement LLINs in December 2014 was followed by significant reduction in monthly malaria incidence in 2015 among children <5 years of age (OR = 0.94, 95% CI 0.91–0.996, P < 0.0001) and those ≥5 years of age (OR = 0.91, 95% CI 0.88–0.94, P < 0.0001)

During the peak months of transmission, malaria incidence reduced from 90 cases/1000 in June 2013 to 50/1000 in June 2015 following introduction of vector control interventions (Fig. 2).

### Reduction in malaria test positivity rate

**Fig. 3:**
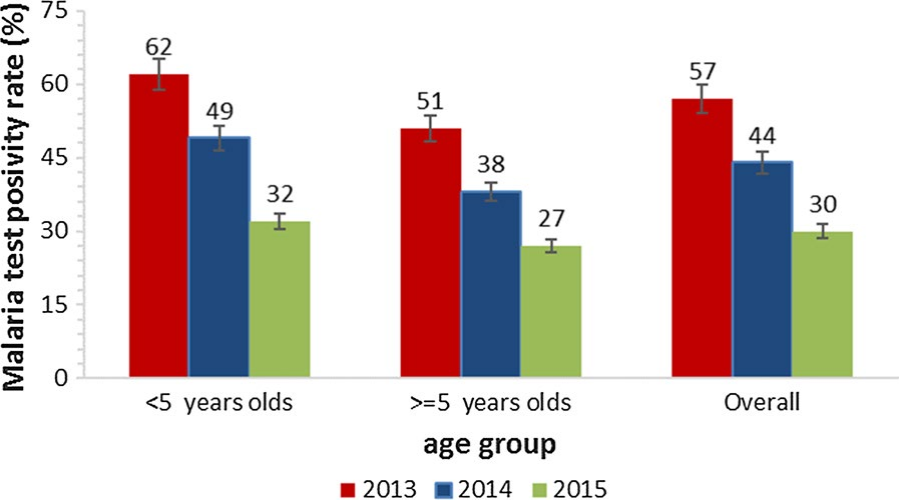
Reduction in malaria test positivity rates in Tororo District 2013–2015. The figure shows overall and age stratified reduction in malaria test positivity rates. The proportions of under five children with parasitologically-confirmed malaria reduced more rapidly than older individuals

The percentage of parasitologically confirmed malaria among individuals tested using RDTs and slide microscopy declined significantly from 57% in 2013 to 30% (Chi = 15, P < 0.0001) in 2015 following introduction of vector control interventions (Fig. 3).

### Change in malaria slide and RDT positivity rates

**Fig. 4:**
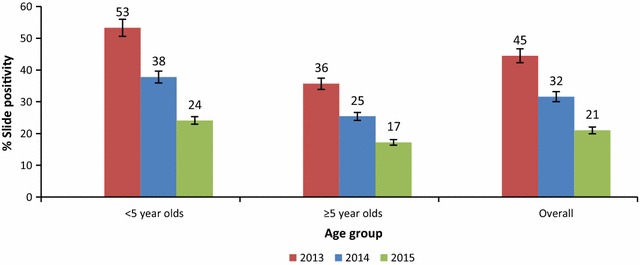
Change in annual malaria slide positivity rates by age group in Tororo District 2012–2015. The figure shows the reduction in slide test positivity rate before intensified vector control interventions were introduced (2012–2013), during the period when universal coverage of communities with LLINs was the only vector control intervention 2014) and when LLINs were combined with IRS in 2015

Overall, slide positivity rate was 45% in 2013 before LLINs were introduced throughout the district, dropped to 32% in 2014 after universal LLIN distribution was achieved and declined further to 21% in 2015 following IRS in combination with LLINs. Similar trends were observed particularly among under five children where slide test positivity rate fell from 53% in 2013 to 24% in 2015 (Fig. 4).

**Fig. 5:**
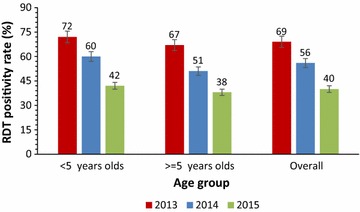
Decline in malaria RDT positivity rates in Tororo District 2013–2015. The figure shows the annual RDT positivity rate 2013–2015

The malaria RDT positivity rate in the district reduced rapidly from 69% in 2013 40% in 2015. A similar trend was observed among children less than 5 years of age (from 72% in 2013 to 42% in 2015) and in individuals aged 5 years and above (from 67% in 2012 to 38%) (Fig. 5).

OPD attendance due to malaria: before universal LLINs (2012–2013), 52% of patients recorded in outpatient registers of health facilities were due to malaria. Following introduction of universal LLINs in 2014, the percentage of malaria cases reported by facilities in outpatient registers reduced to 48% and in 2015 it fell to 30% following implementation of IRS.

## Discussion

This study analysed three malaria burden indicators; incidence, test positivity rate and OPD attendance due to malaria to assess change in the disease magnitude in Tororo District since the introduction of vector control interventions. The findings show consistent reduction in malaria incidence, test positivity rate, and OPD attendance due to malaria following universal LLIN coverage was achieved, but most importantly, after universal LLIN coverage was combined with IRS. This suggests that malaria control interventions that combine LLINs with IRS can be more effective than those that use LLINs alone [36]. Findings also show that during 2014 universal coverage of communities with LLINs was followed by a reduction in malaria incidence, but the monthly reduction trend was not significant. This could suggest that LLINs alone might not change malaria endemic level of a district [37].

The lack of significant reduction in malaria incidence between 2013 and 2014 following the universal distribution of LLINs in Tororo District in 2013 might mean that when LLINs were used alone, there was inadequate protection of communities from malaria vector bites to cut down transmission [12]. This could be explained by several factors. First, during the implementation of universal LLINs, two persons in a household received one net. It was difficult to ascertain whether everyone slept under the same net every night. Second, adherence to LLIN use may have been low, as suggested by data in the Uganda National Malaria Indicator Survey 2014. Third, increasing resistance of vectors to pyrethroid insecticides used in LLINs [7] may have led to reduced effectiveness of the treated nets. Assessing bed net ownership and usage was beyond the scope of this study because relevant data were not captured by the routine surveillance system. Nevertheless, these findings provide more evidence to earlier susceptibility studies [7] which reported failure of LLINs to reduce indoor biting *Anopheles* density and randomized trials which found insignificant malaria reduction when LLINs were used in communities without IRS [36].

The study also found that combining LLINs with IRS was followed by rapid decline in malaria incidence and a significant reduction of the disease in 2015. Indoor residual spraying is known to rapidly reduce malaria vectors and protect communities from infective bites [22] and might have protected all people who did not sleep under LLINs and gave additional protection to those who slept under treated nets [22]. The average house coverage with IRS in Tororo was 90% in 2015, according to a report by the District Health Office [unpublished]. A combination of the two interventions, therefore, might have achieved greater reduction of malaria vectors resulting into significant reduction in malaria transmission.

The decline of malaria in Tororo District is similar to the rapid malaria reduction which IRS and LLINs achieved in Northern Uganda [38]. These findings are also consistent with studies which described LLIN and IRS combination as synergistic malaria control strategies [12, 36]. The reduction observed is in line with the reported progress of malaria reduction in African countries by WHO [3]. However unlike the findings by Jagannathan et al. who found increasing trend of malaria in a cohort study of 100 children following initial vector control using LLINs in Tororo [39], this study found insignificant decrease in malaria incidence following universal distribution of LLINs alone. Results obtained by this analysis might be more reflective of realities in the communities at large because the data used were about the whole district.

Variation in monthly reporting by health facilities (70–99%), missing and incomplete data were among the limitations of this study. Data were only available from July 2012 when DHIS2 was rolled out in the district. We used 2012 to demonstrate the monthly trend before LLINs, but did not compare the data to other years. Inadequate data on other key malaria indicator variables such as mortality rate and malaria in pregnancy limited our analysis to three parameters. There was lack of data 3 years before implementation of vector control interventions as baseline for most accurate descriptive comparison. Assessing demographic and environmental factors which could have contributed to decline in malaria was beyond our scope due to lack of event records. The number of malaria cases used in computing incidence comprised of clinically diagnosed and the laboratory confirmed because the roll out of the test and treat policy in the district was gradual between 2012 and 2015. However, because health workers used same clinical guidelines for diagnosis, the incidence we obtained might give a fairly accurate estimation of the malaria reduction.

## Conclusions

A rapid reduction in malaria incidence was observed in Tororo District following the introduction of IRS in addition to LLINs. There was no significant reduction in malaria incidence following universal distribution of LLINs to communities before introduction of IRS.

## Abbreviations

ACT: artemisinin combination therapy
CDC: Centers for Disease Control and Prevention
DHIS2: District Health Information System version 2
EIR: entomological inoculation rate
HMIS: Health Management Information System
ICCM: Integrated Community Case Management
IRS: indoor residual spraying
LLIN: long-lasting insecticide-treated net
OPD: out patient
PEPFAR: President’s Emergency Plan for AIDS Relief
PMI: President’s Malaria Initiative
RDT: rapid diagnostic test
SPR: slide positivity rate

## References

[1] WHO. World malaria report 2016. Geneva: World Health Organization; 2016.

[2] Gallup JL, Sachs JD (2001). The economic burden of malaria. Am J Trop Med Hyg.

[3] WHO. World Malaria Report 2014. Geneva: World Health Organization; 2014.

[4] Gething PW, Patil AP, Smith DL, Guerra CA, Elyazar IR, Johnston GL (2011). A new world malaria map: *Plasmodium falciparum* endemicity in 2010. Malar J..

[5] Okello PE, Van Bortel W, Byaruhanga AM, Correwyn A, Roelants P, Talisuna A (2006). Variation in malaria transmission intensity in seven sites throughout Uganda. Am J Trop Med Hyg.

[6] Hay SI, Guerra CA, Gething PW, Patil AP, Tatem AJ, Noor AM (2009). A world malaria map: *Plasmodium falciparum* endemicity in 2007. PLoS Med..

[7] Okia M, Ndyomugyenyi R, Kirunda J, Byaruhanga A, Adibaku S, Lwamafa DK (2013). Bioefficacy of long-lasting insecticidal nets against pyrethroid-resistant populations of *Anopheles gambiae* s.s. from different malaria transmission zones in Uganda. Parasit Vectors..

[8] Proietti C, Pettinato DD, Kanoi BN, Ntege E, Crisanti A, Riley EM (2011). Continuing intense malaria transmission in northern Uganda. Am J Trop Med Hyg.

[9] Hay SI, Okiro EA, Gething PW, Patil AP, Tatem AJ, Guerra CA (2010). Estimating the global clinical burden of *Plasmodium falciparum* malaria in 2007. PLoS Med..

[10] Hay SI, Smith DL, Snow RW (2008). Measuring malaria endemicity from intense to interrupted transmission. Lancet Infect Dis..

[11] National Malaria Control Programme. Uganda Malaria Reduction Strategic Plan 2014 – 2020. Kampala, 2014.

[12] Fullman N, Burstein R, Lim SS, Medlin C, Gakidou E (2013). Nets, spray or both? The effectiveness of insecticide-treated nets and indoor residual spraying in reducing malaria morbidity and child mortality in sub-Saharan Africa. Malar J..

[13] National Malaria Control Programme. Uganda malaria quarterly bulletin. In: Malaria quarterly bulletin. Kampala: Ministry of Health; 2014.

[14] Keating J, Locatelli A, Gebremichael A, Ghebremeskel T, Mufunda J, Mihreteab S (2011). Evaluating indoor residual spray for reducing malaria infection prevalence in Eritrea: results from a community randomized control trial. Acta Trop.

[15] English M, Schellenberg J, Todd J (2011). Assessing health system interventions: key points when considering the value of randomization. Bull World Health Organ.

[16] Eisele T, Macintyre K, Eckert E, Beier J, Killeen G. Evaluating malaria interventions in Africa: a review and assessment of recent research. MEASURE Evaluation, Carolina Population Center, University of North Carolina at Chapel Hill; 2000.

[17] Gladwin J, Dixon R, Wilson T (2003). Implementing a new health management information system in Uganda. Health Policy Plan..

[18] Kiberu VM, Matovu JK, Makumbi F, Kyozira C, Mukooyo E, Wanyenze RK (2014). Strengthening district-based health reporting through the district health management information software system: the Ugandan experience. BMC Med Inform Decis Mak.

[19] Cibulskis RE, Bell D, Christophel E-M, Hii J, Delacollette C, Bakyaita N (2007). Estimating trends in the burden of malaria at country level. Am J Trop Med Hyg.

[20] Yeka A, Gasasira A, Mpimbaza A, Achan J, Nankabirwa J, Nsobya S (2012). Malaria in Uganda: challenges to control on the long road to elimination: I. epidemiology and current control efforts. Acta Trop.

[21] Talisuna A, Adibaku S, Dorsey G, Kamya MR, Rosenthal PJ (2012). Malaria in Uganda: challenges to control on the long road to elimination II. The path forward. Acta Trop.

[22] Uganda Bureau of Statistics, USAID. Uganda Malaria Indicator Survey 2009. Kampala: Ministry of Fianance Planning and Economic Development; 2010.

[23] Talisuna A, Langi P, Bakyaita N, Egwang T, Mutabingwa T, Watkins W (2002). Intensity of malaria transmission, antimalarial-drug use and resistance in Uganda: what is the relationship between these three factors?. Trans R Soc Trop Med Hyg.

[24] Pullan RL, Bukirwa H, Staedke SG, Snow RW, Brooker S (2010). Plasmodium infection and its risk factors in eastern Uganda. Malar J..

[25] Wanzirah H, Tusting LS, Arinaitwe E, Katureebe A, Maxwell K, Rek J (2015). Mind the gap: house structure and the risk of malaria in Uganda. PLoS ONE.

[26] Zurovac D, Tibenderana JK, Nankabirwa J, Ssekitooleko J, Njogu JN, Rwakimari JB (2008). Malaria case-management under artemether–lumefantrine treatment policy in Uganda. Malar J..

[27] Nankabirwa J, Zurovac D, Njogu JN, Rwakimari JB, Counihan H, Snow RW (2009). Malaria misdiagnosis in Uganda–implications for policy change. Malar J..

[28] Graz B, Willcox M, Szeless T, Rougemont A (2011). “ Test and treat” or presumptive treatment for malaria in high transmission situations? A reflection on the latest WHO guidelines. Malar J..

[29] WHO Malaria Policy Advisory Committee and Secretariat (2014). Malaria Policy Advisory Committee to the WHO: conclusions and recommendations of fifth biannual meeting (March 2014). Malar J..

[30] Kilian A, Boulay M, Koenker H, Lynch M (2010). How many mosquito nets are needed to achieve universal coverage? Recommendations for the quantification and allocation of long-lasting insecticidal nets for mass campaigns. Malar J..

[31] Beier JC, Keating J, Githure JI, Macdonald MB, Impoinvil DE, Novak RJ (2008). Integrated vector management for malaria control. Malar J..

[32] Kleinschmidt I, Schwabe C, Shiva M, Segura JL, Sima V, Mabunda SJA (2009). Combining indoor residual spraying and insecticide-treated net interventions. Am J Trop Med Hyg.

[33] Ramphul U, Boase T, Bass C, Okedi LM, Donnelly MJ, Müller P (2009). Insecticide resistance and its association with target-site mutations in natural populations of *Anopheles gambiae* from eastern Uganda. Trans R Soc Trop Med Hyg.

[34] Mawejje HD, Wilding CS, Rippon EJ, Hughes A, Weetman D, Donnelly MJ (2013). Insecticide resistance monitoring of field-collected *Anopheles gambiae* s.l. populations from Jinja, eastern Uganda, identifies high levels of pyrethroid resistance. Med Vet Entomol.

[35] Jensen TP, Bukirwa H, Njama-Meya D, Francis D, Kamya MR, Rosenthal PJ (2009). Use of the slide positivity rate to estimate changes in malaria incidence in a cohort of Ugandan children. Malar J..

[36] West PA, Protopopoff N, Wright A, Kivaju Z, Tigererwa R, Mosha FW (2014). Indoor residual spraying in combination with insecticide-treated nets compared to insecticide-treated nets alone for protection against malaria: a cluster randomised trial in Tanzania. PLoS Med..

[37] Smithuis FM, Kyaw MK, Phe UO, van der Broek I, Katterman N, Rogers C (2013). The effect of insecticide-treated bed nets on the incidence and prevalence of malaria in children in an area of unstable seasonal transmission in western Myanmar. Malar J..

[38] Steinhardt LC, Yeka A, Nasr S, Wiegand RE, Rubahika D, Sserwanga A (2013). The effect of indoor residual spraying on malaria and anemia in a high-transmission area of northern Uganda. Am J Trop Med Hyg.

[39] Jagannathan P, Muhindo MK, Kakuru A, Arinaitwe E, Greenhouse B, Tappero J (2012). Increasing incidence of malaria in children despite insecticide-treated bed nets and prompt anti-malarial therapy in Tororo, Uganda. Malar J..

